# Cytoskeletal Expression and Remodeling in Pluripotent Stem Cells

**DOI:** 10.1371/journal.pone.0145084

**Published:** 2016-01-15

**Authors:** Liana C. Boraas, Julia B. Guidry, Emma T. Pineda, Tabassum Ahsan

**Affiliations:** Department of Biomedical Engineering, Tulane University, New Orleans, Louisiana, United States of America; University of Connecticut, UNITED STATES

## Abstract

Many emerging cell-based therapies are based on pluripotent stem cells, though complete understanding of the properties of these cells is lacking. In these cells, much is still unknown about the cytoskeletal network, which governs the mechanoresponse. The objective of this study was to determine the cytoskeletal state in undifferentiated pluripotent stem cells and remodeling with differentiation. Mouse embryonic stem cells (ESCs) and reprogrammed induced pluripotent stem cells (iPSCs), as well as the original un-reprogrammed embryonic fibroblasts (MEFs), were evaluated for expression of cytoskeletal markers. We found that pluripotent stem cells overall have a less developed cytoskeleton compared to fibroblasts. Gene and protein expression of smooth muscle cell actin, vimentin, lamin A, and nestin were markedly lower for ESCs than MEFs. Whereas, iPSC samples were heterogeneous with most cells expressing patterns of cytoskeletal proteins similar to ESCs with a small subpopulation similar to MEFs. This indicates that dedifferentiation during reprogramming is associated with cytoskeletal remodeling to a less developed state. In differentiation studies, it was found that shear stress-mediated differentiation resulted in an increase in expression of cytoskeletal intermediate filaments in ESCs, but not in iPSC samples. In the embryoid body model of spontaneous differentiation of pluripotent stem cells, however, both ESCs and iPSCs had similar gene expression for cytoskeletal proteins during early differentiation. With further differentiation, however, gene levels were significantly higher for iPSCs compared to ESCs. These results indicate that reprogrammed iPSCs more readily reacquire cytoskeletal proteins compared to the ESCs that need to form the network *de novo*. The strategic selection of the parental phenotype is thus critical not only in the context of reprogramming but also the ultimate functionality of the iPSC-differentiated cell population. Overall, this increased characterization of the cytoskeleton in pluripotent stem cells will allow for the better understanding and design of stem cell-based therapies.

## Introduction

Pluripotent stem cells, which include embryonic and induced pluripotent stem cells, have the capacity to self-renew and differentiate to all cells in the body. As a result, these stem cells are an attractive source for cell-based therapies. Effective use for transplantation, however, requires comprehensive understanding of cell traits in order to adequately predict the response *in situ*.

Pluripotent embryonic stem cells are derived from the inner cell mass of a blastocyst and have yet to differentiate into a specialized phenotype. Induced pluripotent stem cells, of particular interest due to their potential for personalized medicine, are instead reprogrammed from adult differentiated cells of various types, ranging from fibroblasts to blood cells [1]. Both types of pluripotent stem cells have similar morphologies and differentiate to the three germ lineages [2,3,4], yet have been found to be dissimilar in other traits, such as methylation patterns [5] and genetic expression [6]. Cell properties not only indicate cell state, but also regulate stem cell fate changes due to microenvironmental factors. Thus, thorough characterization is necessary for fundamental understanding of these cells to maximize their potential future use.

Cells expanded *in vitro* and then transplanted *in vivo* are exposed to abrupt changes in the physical microenvironment. Normal physiological functions (such as structural movement, tissue stiffness, and cellular contraction) impose compressive, tensile, and shear forces on exogenous cells. The response of stem cells to these types of forces can be vital to the efficacy of these cells *in vivo*. Mechanical forces *in vitro* have already been shown to regulate stem cell fate [7], including viability [8] and apoptosis [9]. Differentiation, a property predominantly associated with stem and progenitor cells, has been of particular focus in numerous studies, including studies that have applied tension [10] and compression [11] directly to these cells or varied the stiffness of the underlying substrate [12]. Our own group has found that embryonic stem cells exposed to fluid shear stress differentiate towards the mesodermal lineage [13] and specifically to the endothelial phenotype [14]. The exact intracellular mechanisms that govern these observed mechanoresponses in stem cells, however, have yet to be fully characterized.

The cytoskeleton, an intracellular network of structural proteins, plays a large role in the cellular response to the external microenvironment. This network, composed of microtubules, microfilaments (actin), and intermediate filaments, is complex and extensively developed in many differentiated cells and converts external mechanical forces into intracellular signals. For example, shear stress applied to endothelial cells is transmitted via the cytoskeleton to the nucleus to induce changes in gene expression [15]. In addition, the cytoskeleton can regulate broader aspects of the mechanoresponse, such as constraining swelling of neuronal cells in response to osmotic stress [16]. In iPSCs specifically, hyperosmolarity also induces remodeling of cytoskeletal actin [17]. While the cytoskeleton is understood to be central to the mechanoresponse of cells to external cues, still little is known about the state of the intracellular network in pluripotent stem cells.

The objective of this study was to determine the cytoskeletal state in undifferentiated pluripotent stem cells and their cytoskeletal remodeling with differentiation. Mouse embryonic stem cells (ESCs) and reprogrammed induced pluripotent stem cells (iPSCs), as well as the original un-reprogrammed (parental) mouse embryonic fibroblasts (MEFs) from which the iPSCs were reprogrammed, were evaluated for gene and protein expression of cytoskeletal markers. Cytoskeletal markers were also evaluated after ESC and iPSC differentiation, using either an embryoid body model in 3D suspension culture or shear stress in 2D adherent culture.

## Materials and Methods

### Cell Culture

Pluripotent stem cells (embryonic and induced) and mouse embryonic fibroblasts were cultured using standard techniques. Mouse embryonic stem cells (ESCs; ESD3 from ATCC) and induced pluripotent stem cells (iPSCs; WP5 from STEMGENT; [18]) were expanded, as described previously [19]. Briefly, pluripotent cells were initially expanded on a mitotically inactivated feeder layer and then maintained on gelatin-coated plastic in culture medium, which consisted of Dulbecco’s Modification of Eagles Medium, 15% ESC-qualified fetal bovine serum, 1000 U/ml leukemia inhibitory factor (LIF; EMD Millipore), 2 mM L-glutamine, 0.1 mM non-essential amino acids, and antibiotics. Mouse embryonic fibroblasts (MEFs; CF-1 from ATCC and passaged less than 3 times), the parental cell line from which were generated the iPSCs used in this study, were cultured in Alpha Modification of Eagle’s Medium supplemented with 10% fetal bovine serum and antibiotics before assessment.

### Embryoid Body Differentiation

ESCs and iPSCs were spontaneously differentiated for 8 days as embryoid bodies in 3D suspension culture (ESC-EBs and iPSC-EBs, respectively). Pluripotent stem cells (0.5x10^6^ cells) were placed in non-TC treated dishes in 10 mL of culture medium without LIF and cultured on a rotary shaker (New Brunswick, Enfield, CT; set to 40 RPM) to prevent agglomeration. Culture medium and dishes were changed daily after the second day using gravity separation.

### Application of Shear Stress

Pluripotent cells were differentiated in medium without LIF and exposed to shear stress, as described previously [13,19]. As in those studies, glass slides were coated with collagen type IV (3.5 μg/cm^2^; BD Biosciences, Bedford, MA) for one hour. ESCs and iPSCs were then seeded onto slides at 10,000 cells/cm^2^ and 40,000 cells/cm^2^, respectively, to generate similar cell densities just prior to the application of shear. All samples were cultured for two days in differentiation medium (Minimum Essential Alpha Media, 10% fetal bovine serum, 0.1 mM beta-mercaptoethanol, and antibiotics). Fluid flow was applied to cells on slides using a parallel plate bioreactor system, in which medium was recirculated through a flow chamber with a defined channel geometry. Using a calibrated flow rate, a steady laminar shear stress of 5.0 dynes/cm^2^ was applied for two days (SHEAR). Control samples (STATIC) were cultured under the same time and volume conditions. SHEAR and STATIC samples were imaged and analyzed for gene expression of cytoskeletal proteins.

### Morphological Assessment

Phase contrast microscopy was used to visualize the morphology of cells during culture in two dimensions, with comparisons made between ESCs, iPSCs, and MEFs. Images were also taken of differentiating pluripotent cells as embryoid bodies or on glass slides.

### Immunocytochemistry Images

Immunocytochemistry was used to visualize protein expression in ESCs, iPSCs, and MEFs. Pluripotent cells typically expand as colonies of tightly clustered cells, making it difficult to distinguish *in situ* the protein expression profiles of individual cells (S1 Fig). To isolate protein expression in single cells, colonies were dissociated (Accutase), replated at a low cell density, and allowed to adhere overnight (15–18 hours) prior to immunostaining for protein expression. MEFs were also similarly replated to allow for direct comparison.

Cells were fixed with 4% formaldehyde for 15 min and stored in PBS at 4°C until they were fluorescently stained. Some samples were stained with phalloidin (Molecular Probes) to visualize F-actin. Samples for immunostaining were blocked with serum and then tagged with primary and secondary antibodies. Primary antibodies used were specific to lamin A/C (Lamin A/C Antibody (N-18): sc-6215, goat polyclonal, 1:200, Santa-Cruz), nestin (Nestin 20 PRB-315C, rabbit polyclonal, 1:200, Covance), and vimentin (Vimentin Antibody (FITC) (61R-V104AFT), bovine monoclonal, 1:100, Fitzgerald), while the secondary antibody was conjugated with AF488 (1:200, Molecular Probes). All samples were counterstained with HOECHST 33258 to mark DNA. Images of protein expression were taken using a Nikon A1 confocal microscope.

### Gene Expression

Samples were evaluated for gene expression as described previously [13]. For each sample, 1 μg of RNA was isolated (Qiagen), converted into cDNA (Invitrogen), and analyzed using standard real-time PCR with SYBR® Green on a StepOnePlus PCR System (Applied Biosystems). Primers were designed to assess cytoskeleton expression for skeletal muscle actin (*Acta1)*, smooth muscle actin (*Acta2)*, vimentin (*Vim)*, nestin (*Nes)*, lamin A (*Lmna)*, and tubulin alpha 1b (*Tuba1b*). Gene expression levels were determined using standard curves and reported normalized to expression of glyceraldehyde-3-phosphate dehydrogenase (*Gapdh*).

### Statistical Analysis

Quantitative results are presented as mean ± standard error of the mean (n = 3 independent trials). Comparison between ESC, iPSC, and MEF values were analyzed with a one-way ANOVA, with pair-wise comparisons using a Tukey test as appropriate. Direct comparisons between SHEAR versus STATIC samples and ESC-EB versus iPSC-EB were analyzed using two-tailed Student’s t-tests. Differences were considered statistically significant for p-values < 0.05.

## Results

### Cell Morphology

**Fig 1:**
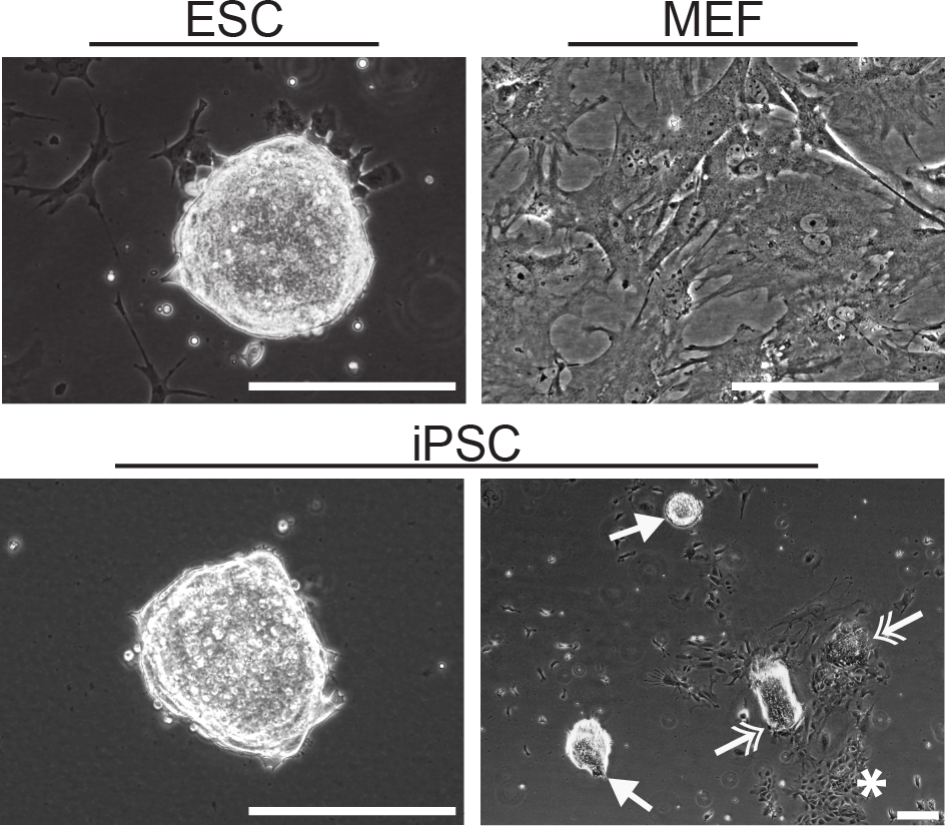
Morphology of ESCs, iPSCs, and MEFs. Representative phase images are shown at both low and high magnification. High magnification images of ESCs show a single highly refractive colony and of MEFs show an adherent spread morphology. A low magnification image of iPSCs shows population heterogeneity, including half dome colonies that were highly refractive (single arrow head; high magnification image also shown), colonies that were less tightly compact with a less refractive outer edge (double headed arrows), and clusters of adherent cells (star). All scale bars represent 200 μm.

Embryonic stem cell (ESC) samples consisted primarily of highly refractive colonies composed of multiple layers of tightly compact cells in a half dome configuration (Fig 1), as is typical of pluripotent cells. Mouse embryonic fibroblasts (MEFs), an adherent phenotype, instead grew with a spread morphology as a single monolayer, as is typical of this phenotype. Induced pluripotent stem cell (iPSC) samples were dominated by colonies similar to ESCs (Fig 1: bottom left and single headed arrows) though some colonies were less tightly compact and had a less refractive outer edge (Fig 1: double headed arrows). In addition, iPSC populations included clusters of cells individually adhered to the culture surface (Fig 1: star). Phenotypic analysis showed ESC and iPSC populations both expressed similar levels of pluripotency markers (S2 Fig). Thus, populations of reprogrammed pluripotent iPSCs, though heterogeneous, are morphologically distinct from their parental somatic MEFs and more similar to pluripotent ESCs.

### Cytoskeletal Gene Expression

**Fig 2:**
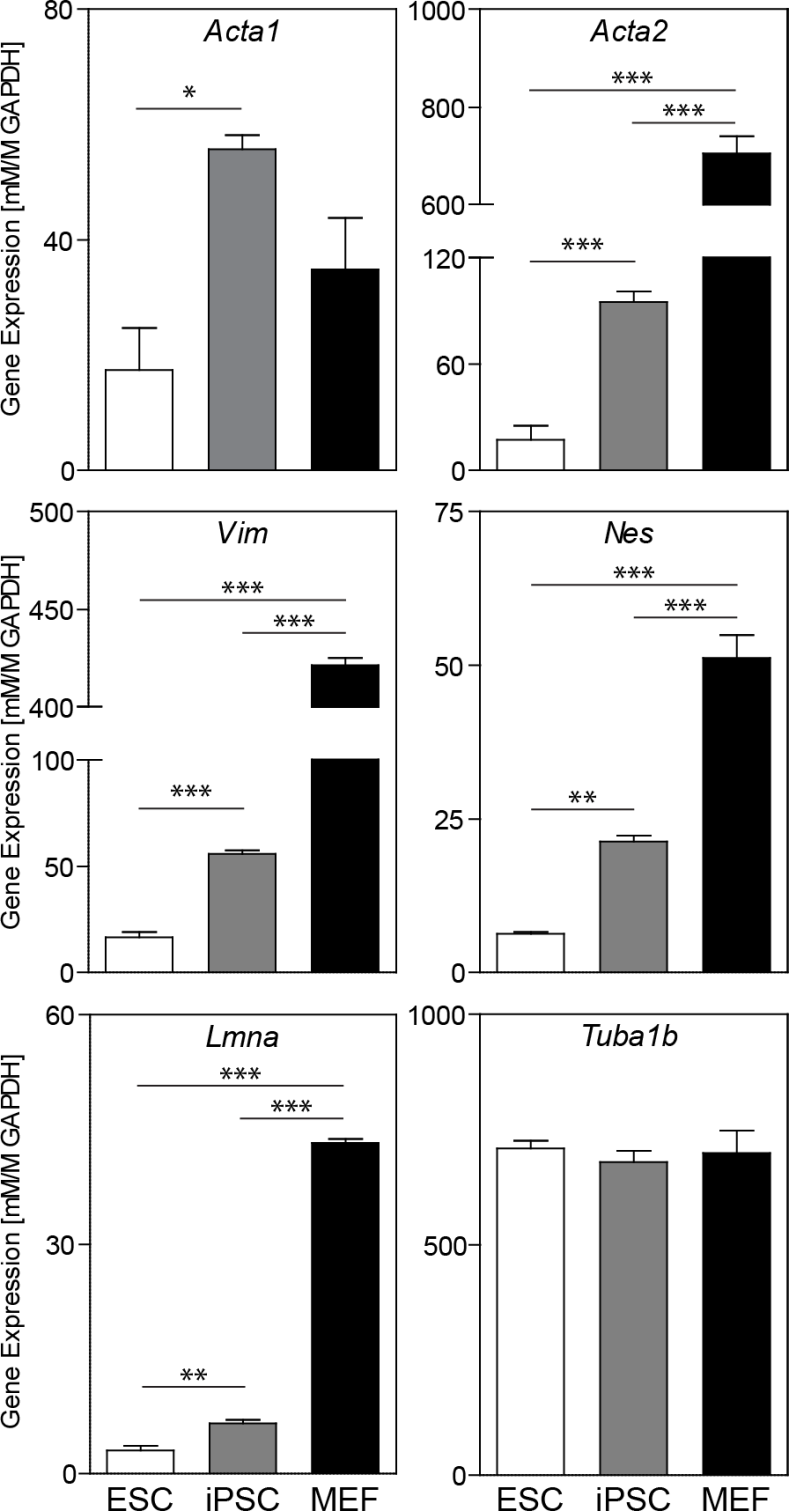
Cytoskeletal gene expression in ESCs, iPSCs, and MEFs. Cytoskeletal gene expression of microfilaments (*Acta1* and *Acta2*), microtubules (*Tuba1b*), and both cytoplasmic (*Vim* and *Nes*) and nuclear (*Lmna*) intermediate filaments are shown (all normalized to *Gapdh*). Data presented are mean ± SEM (n = 3), with significant differences indicated using asterisks (* for p<0.05, ** for p<0.01, and *** for p<0.001).

Cytoskeletal gene expression for actin filaments varied for ESC, iPSC, and MEF populations (Fig 2). Actin expression was evaluated with the *Acta1* and *Acta2* genes for skeletal and smooth muscle actin, respectively. One-way ANOVA analysis indicated a significant difference across ESCs, iPSCs, and MEFs for both *Acta1* (p<0.05) and *Acta2* (p<0.001) expression. Subsequent pair-wise comparisons for *Acta1* indicated significance only in the higher (p<0.05) level for iPSCs compared to ESCs. As a marker for the actin specific to the skeletal muscle cell phenotype, the slight difference in the *Acta1* gene expression levels observed among these cells is not likely to be biologically meaningful. Levels of *Acta2*, a marker of the more ubiquitous smooth muscle cell actin, were instead significantly and markedly different for all comparisons between phenotype. *Acta2* expression levels in MEFs were higher than both ESCs (40X, p<0.001) and iPSCs (7X, p<0.001), though levels for iPSCs were still notably elevated compared to ESCs (95±6 vs 17±8, p<0.001). Thus, both pluripotent stem cell types have markedly lower levels of gene expression for smooth muscle cell actin compared to MEFs, with iPSCs still having a higher expression level than ESCs.

Gene expression for both cytoplasmic (*Vim* and *Nes*) and nuclear (*Lmna*) intermediate filaments, as well as tubulin, was also evaluated (Fig 2). One-way ANOVA analysis for each intermediate filament resulted in a significant difference across the cell phenotypes (p<0.001 in each case). *Vim* and *Lmna* expression in MEFs was more than an order of magnitude greater compared to ESCs (25- and 14-fold changes, respectively), with iPSC levels significantly elevated but only 2-3X times that of ESCs (significance levels indicated in Fig 2). Changes in *Nes* expression after reprogramming were more modest, where levels in iPSCs were both 3X less than MEFs (p<0.001) and 3X greater than ESCs (p<0.01). No difference in *Tuba1b* expression, a marker of microtubules, was detected across the different cell types. This last finding was not unexpected as microtubules are fundamentally necessary for cell survival [20,21]. Overall, expression levels of intermediate filaments, similar to smooth muscle cell actin, were markedly higher in MEFs compared to either pluripotent stem cell type, with levels higher in iPSCs compared to ESCs. Taken together, these gene expression changes indicate that some cytoskeletal remodeling is associated with reprogramming to the pluripotent state.

### Cytoskeletal Protein Expression

**Fig 3:**
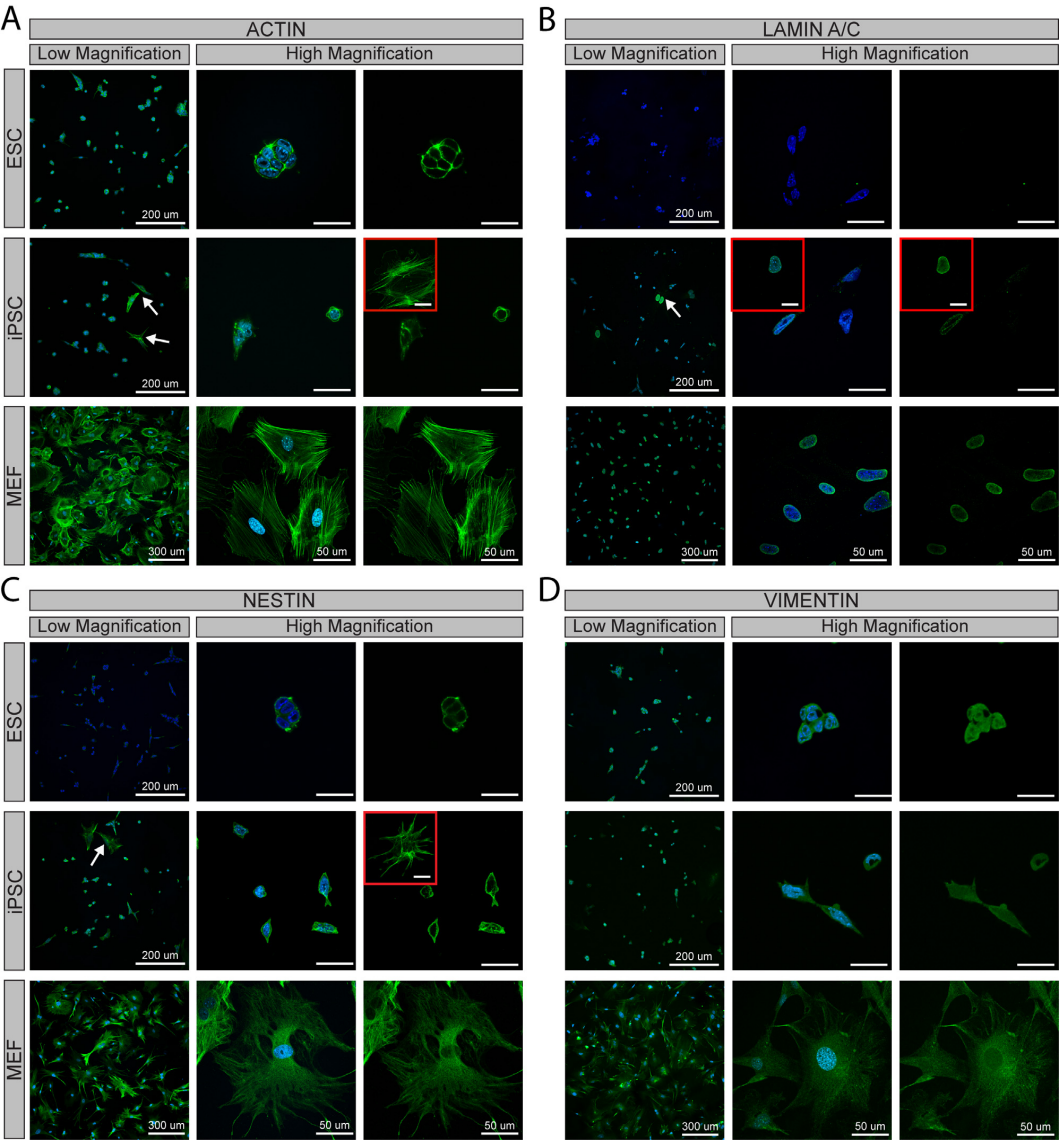
Immunofluorescent staining for cytoskeletal proteins in ESCs, iPSCs, and MEFs. Samples were stained for ACTIN **(A)**, LAMIN A/C **(B)**, NESTIN **(C)** and VIMENTIN **(D)** (cytoskeletal elements in green) with a nuclear counterstain (blue). Shown are images at low magnification (merged: left columns) and high magnification (merged: middle columns; cytoskeleton only: right columns). Single headed arrows and inserts highlight a subpopulation of cells with a spread morphology in iPSC cultures. Scale bars = 30 μm unless otherwise specified.

Cytoskeletal protein expression patterns for microfilaments (ACTIN) were distinct between replated cultures of MEFs, iPSCs, and ESCs (Fig 3). MEF cultures stained intensely for ACTIN throughout the spread cell bodies (Fig 3A), with distinct stress fibers observable under high magnification. ESCs, smaller in size compared to MEFs, overall had less observable protein expression of ACTIN. High magnification images showed that the ACTIN expressed in ESCs, unlike MEFs, was concentrated in the compact cytoplasm around the nucleus and did not spread to form stress fibers. Cultures of iPSCs included a few cells with a spread MEF-like ACTIN expression (Fig 3A: indicated by arrows and shown in insert) but contained mostly cells with low protein expression similar to ESC cultures. As observed in ESCs, high magnification images revealed ACTIN expression in these iPSCs was tightly compacted in the cytoplasm surrounding the nucleus. Thus, MEFs and ESCs had distinct arrangements of ACTIN expression, while iPSC cultures were comprised of cells with both MEF-like and ESC-like ACTIN expression patterns.

Protein expression of both nuclear (LAMIN A/C) and cytoplasmic (NESTIN and VIMENTIN) intermediate filaments was also evaluated (Fig 3B–3D). As expected, LAMIN A/C expression in MEF cultures was restricted to the nucleus, with faint expression throughout and strong expression along the edges (Fig 3B). In contrast, LAMIN A/C expression in ESCs was not detected. Cultures of iPSCs contained cells with MEF-like LAMIN A/C expression (Fig 3B: indicated by an arrow and shown in insert), as well as cells with little to no detectable expression. NESTIN was expressed throughout the cytoplasm in MEFs and concentrated around the nucleus in ESCs (Fig 3C). In a trend similar to that found with ACTIN and LAMIN A/C, NESTIN patterns in iPSC cultures consisted of a few cells with MEF-like expression (Fig 3C: single headed arrows and insert) but was dominated by cells with ESC-like expression. Cytoplasmic VIMENTIN, on the other hand, was expressed throughout the cell in all three populations with visual differences seemingly only due to the smaller size of ESCs and some iPSCs (Fig 3D). Overall, cytoskeletal protein expression patterns were distinct for ESCs and MEFs, with iPSC cultures mostly consisting of cells similar to ESCs with a small distinct MEF-like population (Fig 3: arrows, inserts).

### Embryoid Body Differentiation

**Fig 4:**
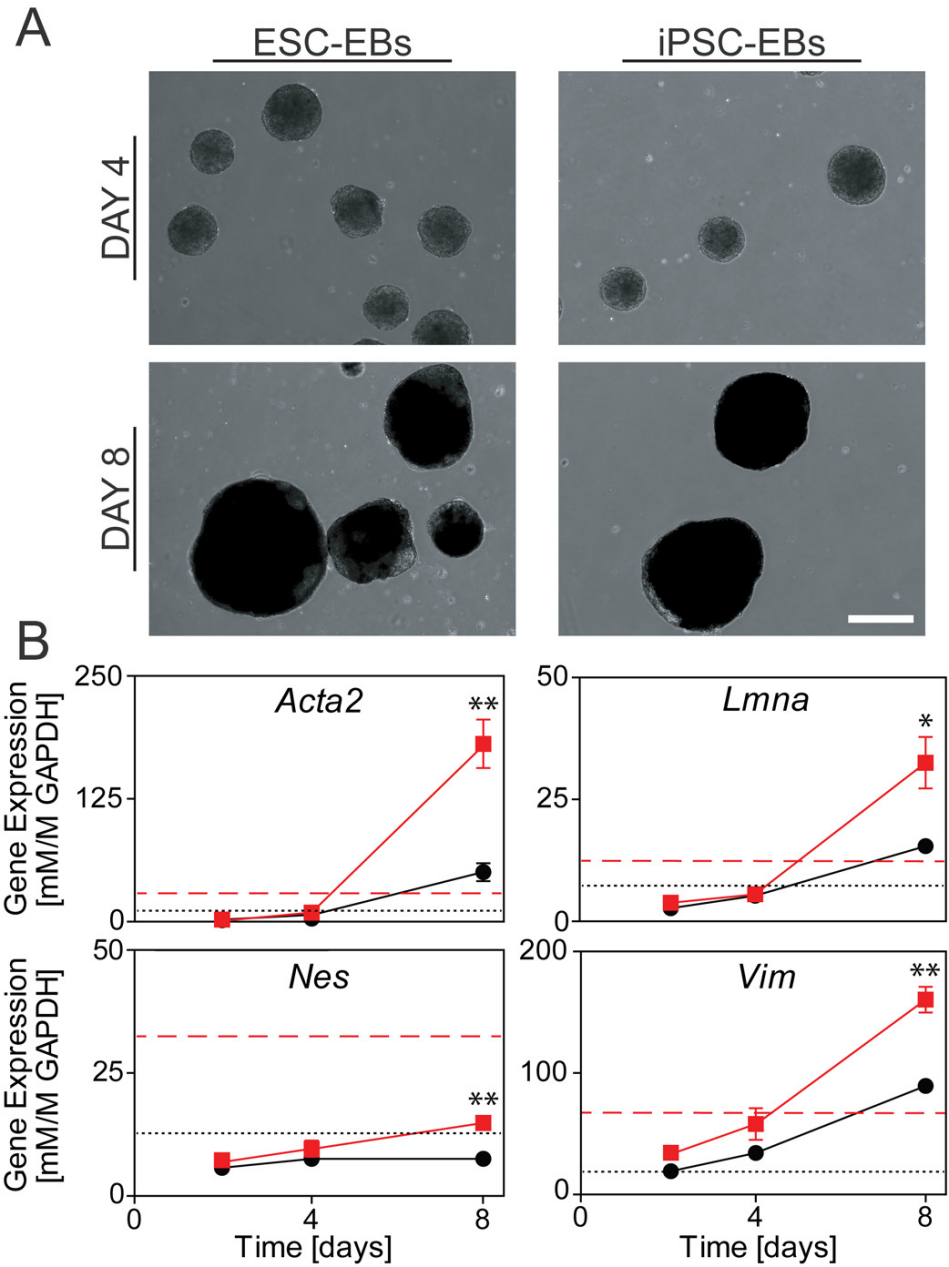
Morphology and cytoskeletal gene expression of differentiating ESCs and iPSCs in the EB model. (**A**) Phase images at the same magnification for ESC-EBs and iPSC-EBs at Day 4 and 8. Scale bar represents 200 μm. (**B**) Gene expression at Day 2, 4, and 8 of *Acta1*, *Vim*, *Nes*, and *Lmna* (all normalized to *Gapdh*) of ESC-EBs (red squares) and iPSC-EBs (black circles). Data are presented as mean ± SEM (n = 3), where significant differences are indicated with asterisks (*p<0.05, **p<0.01). For reference, mean values of expression for undifferentiated ESCs (dotted red line) and iPSCs (dashed black line) from Fig 2 are indicated.

Embryoid Bodies (EBs) were generated to compare differences in cytoskeletal remodeling between spontaneously differentiating ESCs and iPSCs. EBs were cultured for up to 8 days and evaluated for morphology and cytoskeletal gene expression. Phase images revealed that EBs generated from ESCs (ESC-EBs) and iPSCs (iPSC-EBs) had a similar rounded morphology at early timepoints, with both ESC-EBs and iPSC-EBs increasing in size with time (Fig 4A). In this 3D spontaneous differentiation model, gene expression for both cytoplasmic (*Vim* and *Nes*) and nuclear (*Lmna*) intermediate filaments, as well as smooth muscle actin (*Acta2*), increased over time in EBs generated from either cell type (Fig 4B). The increase with time of cytoskeletal expression in ESC-EBs seen in this study is consistent with similar results we published previously [22].

In comparing expression in EBs derived from the two pluripotent stem cell types, levels of all four tested genes in Day 2 and Day 4 samples were not significantly different between ESC-EBs and iPSC-EBs and were similar or below levels in undifferentiated ESCs and iPSCs (Figs 2 and 4B: mean values represented with black dashed and red dotted lines, respectively). The apparent slight drop in certain gene levels between undifferentiated iPSCs and Day 2 iPSC-EBs may reflect the loss of an adherent subpopulation that did not aggregate into EBs. All four genes were higher in EBs derived from reprogrammed iPSCs after 8 days of differentiation. *Acta2* expression in iPSC-EBs was 3.6X greater than ESC-EBs (p<0.01) with consistent differences in the gene expression of the intermediate filaments *Vim* (1.8X, p<0.01), *Nes* (2.0X, p<0.01), and *Lmna* (2.1X, p<0.05). While iPSC-EBs and ESC-EBs have similar morphology and cytoskeletal gene expression patterns during the early stages of differentiation, the acquired expression of cytoskeletal genes was ultimately higher in reprogrammed cells compared to naïve pluripotent stem cells.

### Shear Induced Differentiation

**Fig 5:**
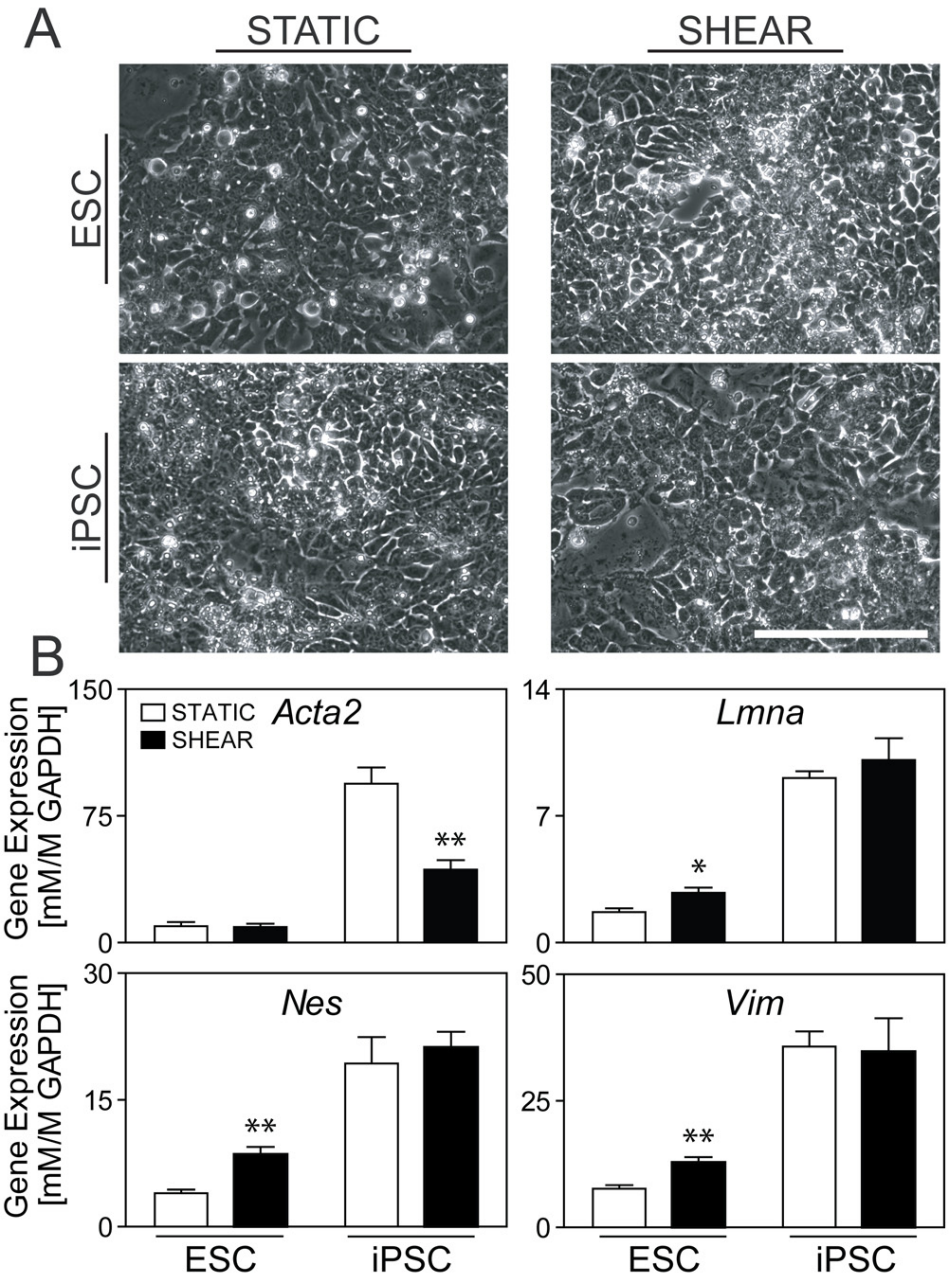
Morphology and cytoskeletal gene expression of ESCs and iPSCs after shear-mediated differentiation. Evaluation of ESCs and iPSCs after SHEAR treatment or as STATIC controls. **(A)** Phase images of all four groups. Scale bar represents 200 μm. **(B)** Gene expression levels for *Acta1*, *Vim*, *Nes*, and *Lmna* (all normalized to *Gapdh*) for STATIC (white bars) and SHEAR (black bars) samples. Data are presented as mean ± SEM (n = 5 for ESCs and n = 3 for iPSCs), where significant differences are indicated with asterisks (*p<0.05, **p<0.01).

We used our established model of applied fluid shear stress [13,14,19] to determine cytoskeletal remodeling in pluripotent stem cells in response to mechanically-driven differentiation. Pluripotent cells were differentiated under either static (STATIC) or steady laminar shear stress (SHEAR) conditions and, as for the EB differentiation study, evaluated for morphology and gene expression of cytoskeletal elements. In previous publications, we have shown that differentiation in 2D is less dynamic compared to 3D [22], but that applied shear stress promotes differentiation to the mesodermal lineage [13,14,19] and the endothelial phenotype in particular [13,14,19]. In these studies, phase images revealed no morphological differences across either cell type or treatment condition. For all samples, differentiation of four days resulted in a layer of spread and attached cells, a configuration distinct from the tight colonies of rounded cells in the initial undifferentiated populations (Fig 5A). Exposure to an applied shear stress of 5.0 dynes/cm^2^ induced an upregulation of *Flk1* (a mesodermal differentiation marker) in the iPSC samples compared to controls (S3 Fig), similar to the differentiation response we have seen previously in ESCs [14]. In terms of cytoskeletal remodeling, however, shear stress had no detectable difference in *Acta2* expression in ESCs (Fig 5B). Shear stress in ESCs did, however, significantly upregulate gene expression of the intermediate filaments *Vim* (1.7X, p<0.01), *Lmna* (1.7X, p<0.05), and *Nes* (2.1X, p<0.01). Similar results were also observed after the application of 1.5 dynes/cm^2^ of shear stress (S4 Fig). In contrast, the application of shear stress to iPSC populations resulted in no detectable change in gene expression of the same intermediate filaments and a downregulation of *Acta2* (55%, p<0.01) compared to iPSC-STATIC controls. These findings indicate that applied fluid shear stress induces cytoskeletal remodeling in pluripotent stem cells but that ESC and iPSC samples respond differently.

## Discussion

In these studies we found that pluripotent stem cells overall have a less developed cytoskeleton compared to fibroblasts. With the exception of a phenotype-specific marker (skeletal muscle actin) and a fundamental cell survival protein (tubulin), gene expression of cytoskeletal markers (smooth muscle cell actin, vimentin, lamin A, and nestin) were all markedly lower for embryonic stem cells (ESCs) compared to mouse embryonic fibroblasts (MEFs). Furthermore, at the protein level, actin stress fibers and nuclear lamin A/C expression were both absent in ESCs but present in MEFs. The lack of an extensive cytoskeletal network in ESCs is consistent with their small rounded morphology in contrast to the spread morphology of MEFs. In addition, induced pluripotent stem cell (iPSC) samples that were reprogrammed from the same MEFs used in this study, had cytoskeletal gene expression at intermediate levels between the two other phenotypes with an observed protein pattern indicating a heterogeneous population of ESC-like and MEF-like cells.

Differentiation, either mechanically-driven by applied shear stress or spontaneous as in embryoid bodies, revealed differences in ESCs and iPSCs in terms of cytoskeletal remodeling. Shear stress applied to ESCs, previously shown to promote mesodermal differentiation [13,14,19], resulted in an increase in expression of intermediate filaments. In iPSC cultures, a similar differentiation response seems to occur (S3 Fig), but there was no similar increase in intermediate filament expression. The presence of a subpopulation of MEF-like cells may account for the already relatively high levels of cytoskeletal gene expression and an inability to detect changes in the iPSC samples with applied stress. In the spontaneous model of differentiation, however, only those cells that aggregate in suspension to form embryoid bodies are maintained in culture and evaluated. Initiation of this model likely selects for primarily the pluripotent stem cells and here it was observed that cytoskeletal expression at early time points in iPSC samples was not detectably different from expression in ESCs. Once these pluripotent stem cells were further differentiated to Day 8, however, gene levels were significantly and markedly different for cytoskeletal markers between iPSCs and ESCs. These longer range differences in cytoskeletal remodeling indicate that the pluripotent cells in these MEF-derived iPSC samples may more readily reacquire cytoskeletal proteins compared to the ESCs that need to form the network *de novo*.

The set of cytoskeletal proteins evaluated in these studies were chosen to include microfilaments, intermediate filaments, and microtubules. For the microfilaments, changes in gene expression patterns across the cell types were not consistent. *Acta2* was expressed in MEFs at levels markedly higher than the pluripotent stem cells, while *Acta1* expression did not have a clear distinction. This was likely because *Acta1* is specific to skeletal muscle cells and *Acta2*, despite its moniker as smooth muscle cell actin, is expressed in various differentiated cells including MEFs. On the protein level, MEFs also contained well-formed actin stress fibers that were not found in the pluripotent stem cells. In contrast, expression of *Tuba1b* did not differ across cell types, which was not unexpected as microtubules are necessary for general cell functions such as cell division, centrosome structure, motor protein transport, and cell structure [20,21]. The expression patterns of the intermediate filaments, however, were similar to that of *Acta2*, with markedly higher expression in MEFs compared to both ESCs and iPSCs. In the case of nuclear lamin A/C, this is consistent with previous findings that expression was absent in pluripotent stem cells and acquired with differentiation [23]. Nestin, predominantly expressed by neural phenotypes, and vimentin, ubiquitously expressed in mesodermal phenotypes, were both found to have much lower levels of expression in the pluripotent stem cells compared to the MEFs. Taken together, these results indicate that not only do embryonic pluripotent stem cells have a less developed cytoskeleton than MEFs, but that in reprogramming of MEFs to iPSCs the cytoskeleton is remodeled to a less developed state in the resulting iPSCs.

Differences in the cytoskeletal network in iPSCs compared to the parental MEFs is consistent with other studies related to dedifferentiation and reprogramming. Others have shown reprogramming requires cells undergo a mesenchymal to epithelial transition, which is associated with reorganization of the cytoskeleton [24]. Remodeling of the cytoskeleton may even be a key rate limiting step in reprogramming to pluripotency. Lacking lamin A, destabilizing actin, or perturbing actin-myosin interactions in MEFs during reprogramming was found to increase the overall efficacy of the reprogramming process [25] [26]. Similarly, knockdown of TESK1, an enzyme related to actin filament stabilization, also increased reprogramming efficacy [27]. Thus, strategies to improve the overall efficiency and efficacy of the reprogramming process may include a focus on the cytoskeleton, perhaps using small molecule inhibitors or mechanical forces to disrupt network stability or even selecting parental phenotypes with little initial cytoskeletal structure.

In these studies the iPSC samples were heterogeneous populations, consisting mostly of cells similar to ESCs but with a small distinct MEF-like population. Based on morphology and protein patterning, it is likely that not all cells were fully reprogrammed with some cells remaining highly similar to the parental MEFs. This heterogeneity would skew the parametric evaluation of gene expression for the entire population and obfuscate the observed effects on changes in gene expression due to shear stress in an adherent system. In the case of the EB differentiation model, which selects for pluripotent stem cells, however, the gene levels during early differentiation were not detectably different between the ESCs and iPSCs. This indicates that during early differentiation the pluripotent cells in the ESC and iPSC samples have similar cytoskeletal expression.

Heterogeneous cell populations in iPSC samples are currently due to a still inefficient process of reprogramming [28]. In our studies, we also found that iPSC samples consisted of both ESC-like and MEF-like subpopulations with distinct protein expression patterns for cytoskeletal elements. Expression of specific cytoskeletal proteins is often used as a marker of differentiated phenotypes [29,30,31] and may be a useful trait to help generate homogeneous cell populations after reprogramming. Gene or protein expression for smooth muscle cell actin, vimentin, nestin, and lamin A was found to be higher in MEFs than pluripotent stem cells. These cytoskeletal proteins may then function as biomarkers to select or deselect for pluripotent stem cells, particularly in the common circumstances where pluripotent stem cells are cultured atop a MEF feeder layer. Sorting using FACS or MACS systems that use antibody-based approaches, however, would be destructive due to the intracellular localization of cytoskeletal proteins. It may instead be more useful to sort based on an attribute that reflects different cytoskeletal states, such as cell stiffness which is known to be dependent on the cytoskeleton [32] and has been shown to be lower in iPSCs compared to their somatic parental cells [33]. Such sorting techniques may include non-destructive direct testing of cell stiffness [34] or the use of pressure-driven microfluidic arrays with elution paths with subcellular cross-sectional areas [35]. Thus, cytoskeletal-based strategies for cell sorting may be helpful in generating homogenous populations of induced pluripotent stem cells.

Fully reprogrammed pluripotent cells seem to have a cytoskeleton similar to pluripotent embryonic cells. This is indicated by a subpopulation of cells in the iPSC samples that have a morphology and protein expression pattern similar to the ESCs. This is further corroborated by similar gene expression levels in the early ESC- and iPSC-derived EB cultures, which select for the pluripotent stem cells. There may be subtle differences, however, that remain between the cytoskeletal state of fully reprogrammed iPSCs and ESCs. While ESC-EB and iPSC-EB samples had similarly low levels of cytoskeletal expression during early differentiation, by Day 8 iPSC-EBs had acquired higher gene expression levels of cytoskeletal filaments. As the iPSCs previously had a robust cytoskeleton, residual parental properties may lead to the potential to regain cytoskeletal expression at a faster rate as compared to the naïve ESCs. Similar differences between ESCs and iPSCs have been found in other properties, including methylation signatures [36], genetic expression [5] [6], actin regulation [37], and viscoelastic properties [38]. These discrepancies, including possible cytoskeletal differences, between ESCs and iPSCs may in part be responsible for variation in their redifferentiation efficiencies [39,40] and terminal cell functionalities [41].

Retained residual properties after reprogramming make the selection of the parental phenotype an important factor in the long-term functionality of an iPSC population. In studies that reprogrammed fibroblasts and blood cell populations, it was found that each re-differentiated more efficaciously to their original phenotype [1]. Furthermore, comparisons of iPSCs derived from either fibroblasts or cord blood were initially indistinguishable in expression of pluripotency markers, but still retained a bias to differentiate towards the parental cell type even in the presence of signals toward alternative cell phenotypes [42]. Fibroblasts and blood cells have very different cytoskeletons and these redifferentiation outcomes after reprogramming may in part be due to residual cytoskeletal properties. In the case of cardiomyocytes, in which functionality is strongly linked to an extensively developed and active cytoskeleton, iPSCs from cardiac progenitor cells compared to fibroblasts from the same donor were indeed more efficient at acquiring sarcomeric alpha-actinin structures [43]. These results indicate that strategic selection of the parental phenotype might be based not only on the differentiated phenotype but also the mechanical properties of iPSC-derived cells, such as cell stiffness or mechanoresponsiveness.

Many emerging cellular therapeutic approaches are based on pluripotent stem cells, especially iPSCs that allow for personalized medicine. Efficacy of these approaches is dependent on understanding the *in vivo* and *in vitro* cellular mechanoresponse, which is regulated by the cytoskeleton. This study found that pluripotent stem cells compared to fibroblasts have markedly lower gene and protein expression for a wide range of cytoskeletal elements. Thus pluripotent cells compared to many differentiated phenotypes would have strikingly different responses to the physical microenvironment *in vivo* and *in vitro*. In addition, we found that reprogramming fibroblasts to the pluripotent state is associated with remodeling towards a much less developed cytoskeletal network. While there is some evidence by others that destabilizing the cytoskeletal network may benefit reprogramming efficiency, systematic studies are still needed to establish a causal relationship. In addition, there are indications in our studies that reprogrammed pluripotent stem cells may retain some residual properties that facilitate the reacquisition of cytoskeletal proteins. The strategic selection of the parental phenotype is thus critical not only in the context of reprogramming but also the ultimate functionality of the iPSC-differentiated cell population. Overall, the increased characterization of the cytoskeleton in pluripotent stem cells in these studies will allow for the better understanding and design of stem cell-based therapies.

## Supporting Information

S1 Fig Actin expression in ESC colonies. Whole ESC colonies were stained with phalloidin and HOECHST 33258 to indicate F-actin and DNA, respectively. Visualization of ACTIN within cells was difficult to discern. For subsequent analyses colonies were disassociated and cells replated for staining.
(TIF)

S2 Fig Expression of pluripotency markers in ESCs and iPSCs. (A) Gene expression for *Nanog*, *Oct4*, and *Sox2* was evaluated in ESCs and iPSCs. No significant difference in *Nanog* expression was detected between cell types however, *Oct4* and *Sox2* expression levels were significantly lower in iPSCs (*, p<0.05 and **, p<0.01). Data presented as mean ± SEM (n = 3). (B) Samples for flow cytometry were prepared from ESC or iPSC cell colonies dissociated with 0.05% Trypsin-EDTA and fixed in 4% formaldehyde. Primary antibodies used were specific to NANOG (Abcam), OCT3/4 (Santa-Cruz) and SOX2 (eBiosciences) while the secondary antibody was conjugated with AF488 (Molecular Probes). Flow cytometry samples were detected with a BD FACSCanto II system where a secondary only control is shown in gray. ESC and iPSC populations had very similar NANOG and OCT3/4 protein expression profiles however, iPSCs expressed lower levels SOX2 compared to ESCs. This data in conjunction with morphological and immunocytochemistry data indicate that the iPSC samples are a heterogeneous population, potentially including cells that are not fully reprogrammed. These pluripotency results indicate, however, that ESC and iPSC samples overall express similar levels of pluripotency markers.
(TIF)

S3 Fig FLK1 gene expression in STATIC and SHEAR iPSCs. Samples were cultured under static conditions for two days and then exposed to two days of either STATIC or SHEAR treatment. Exposure to SHEAR upregulated FLK1 (**, p<0.01), an early mesodermal marker, in iPSCs, similar to the differentiation response we have seen previously in ESCs [13,14]. This suggests that iPSCs and ESCs may have a similar differentiation response under the application of laminar shear stress.
(TIF)

S4 Fig Cytoskeletal Expression with Shear Stress Magnitudes. Pluripotent cells were exposed to either 1.5 or 5.0 dynes/cm^2^ of shear stress and evaluated for cytoskeletal gene expression to determine if there was a magnitude dependent response for either cell type. Cytoskeletal remodeling in response to shear stress was independent of magnitude in ESCs where Vim expression was significantly upregulated in SHEAR samples (p<0.01 for both magnitudes) while no detectable difference was found in *Acta2* expression. Similarly in iPSCs, no detectable difference was found in Vim expression between shear stress magnitude and its STATIC control. *Acta2* expression in iPSCs was not significantly different after 1.5 dynes/cm^2^ of shear stress but downregulated with 5.0 dynes/cm^2^ of shear stress (**, p<0.01).
(TIF)
