# Supplementary figures and images for: Cytoskeletal Expression and Remodeling in Pluripotent Stem Cells
Source: PLoS One. 2016 Jan 15;11(1):e0145084. doi: 10.1371/journal.pone.0145084 (PMC4714815; doi:10.1371/journal.pone.0145084)

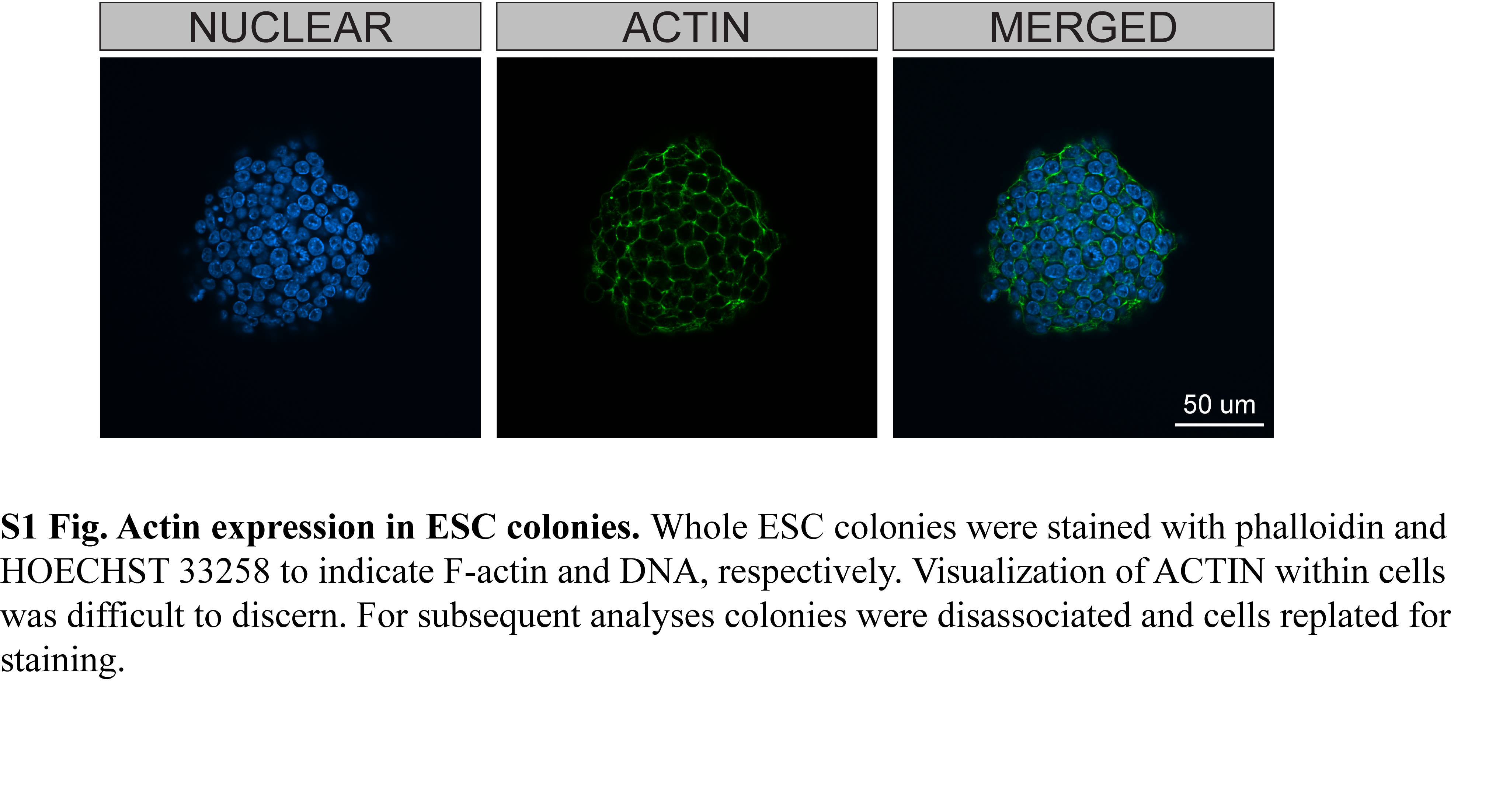

Supplement: S1 Fig — Whole ESC colonies were stained with phalloidin and HOECHST 33258 to indicate F-actin and DNA, respectively. Visualization of ACTIN within cells was difficult to discern. For subsequent analyses colonies were disassociated and cells replated for staining. (TIF) [file pone.0145084.s001.tif]

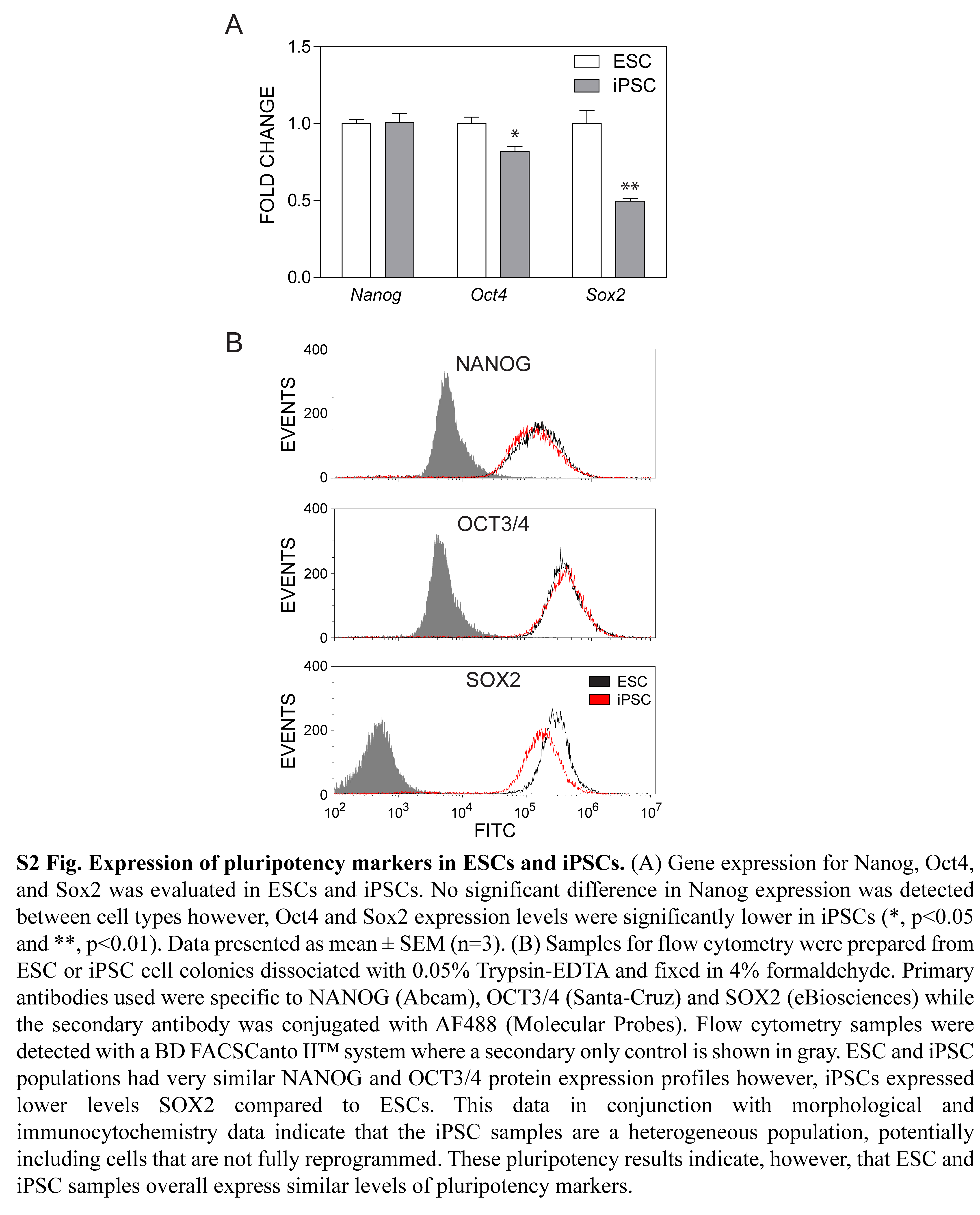

Supplement: S2 Fig — (A) Gene expression for Nanog, Oct4, and Sox2 was evaluated in ESCs and iPSCs. No significant difference in Nanog expression was detected between cell types however, Oct4 and Sox2 expression levels were significantly lower in iPSCs (*, p<0.05 and **, p<0.01). Data presented as mean ± SEM (n = 3). (B) Samples for flow cytometry were prepared from ESC or iPSC cell colonies dissociated with 0.05% Trypsin-EDTA and fixed in 4% formaldehyde. Primary antibodies used were specific to NANOG (Abcam), OCT3/4 (Santa-Cruz) and SOX2 (eBiosciences) while the secondary antibody was conjugated with AF488 (Molecular Probes). Flow cytometry samples were detected with a BD FACSCanto II system where a secondary only control is shown in gray. ESC and iPSC populations had very similar NANOG and OCT3/4 protein expression profiles however, iPSCs expressed lower levels SOX2 compared to ESCs. This data in conjunction with morphological and immunocytochemistry data indicate that the iPSC samples are a heterogeneous population, potentially including cells that are not fully reprogrammed. These pluripotency results indicate, however, that ESC and iPSC samples overall express similar levels of pluripotency markers. (TIF) [file pone.0145084.s002.tif]

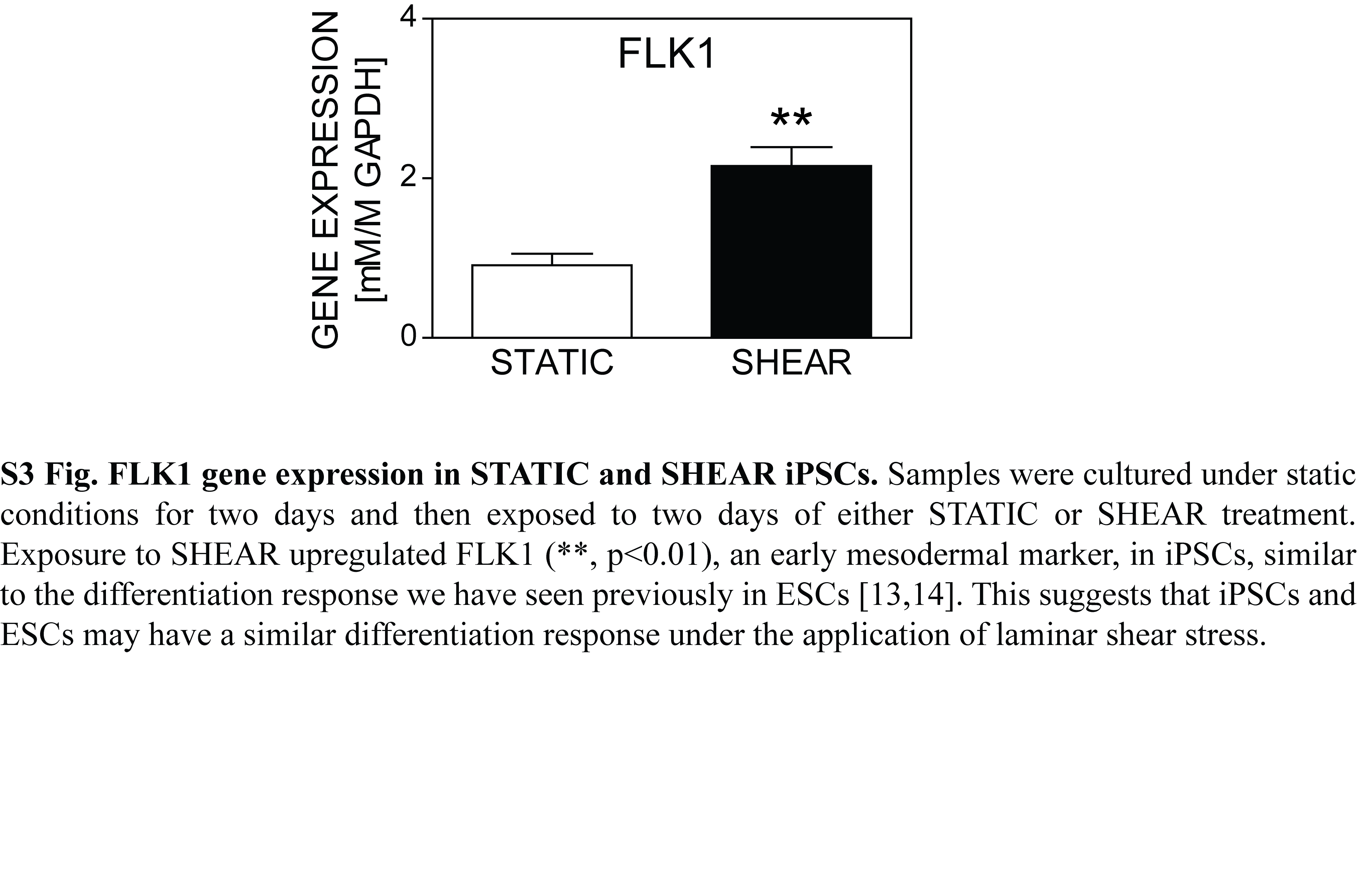

Supplement: S3 Fig — Samples were cultured under static conditions for two days and then exposed to two days of either STATIC or SHEAR treatment. Exposure to SHEAR upregulated FLK1 (**, p<0.01), an early mesodermal marker, in iPSCs, similar to the differentiation response we have seen previously in ESCs [13,14]. This suggests that iPSCs and ESCs may have a similar differentiation response under the application of laminar shear stress. (TIF) [file pone.0145084.s003.tif]

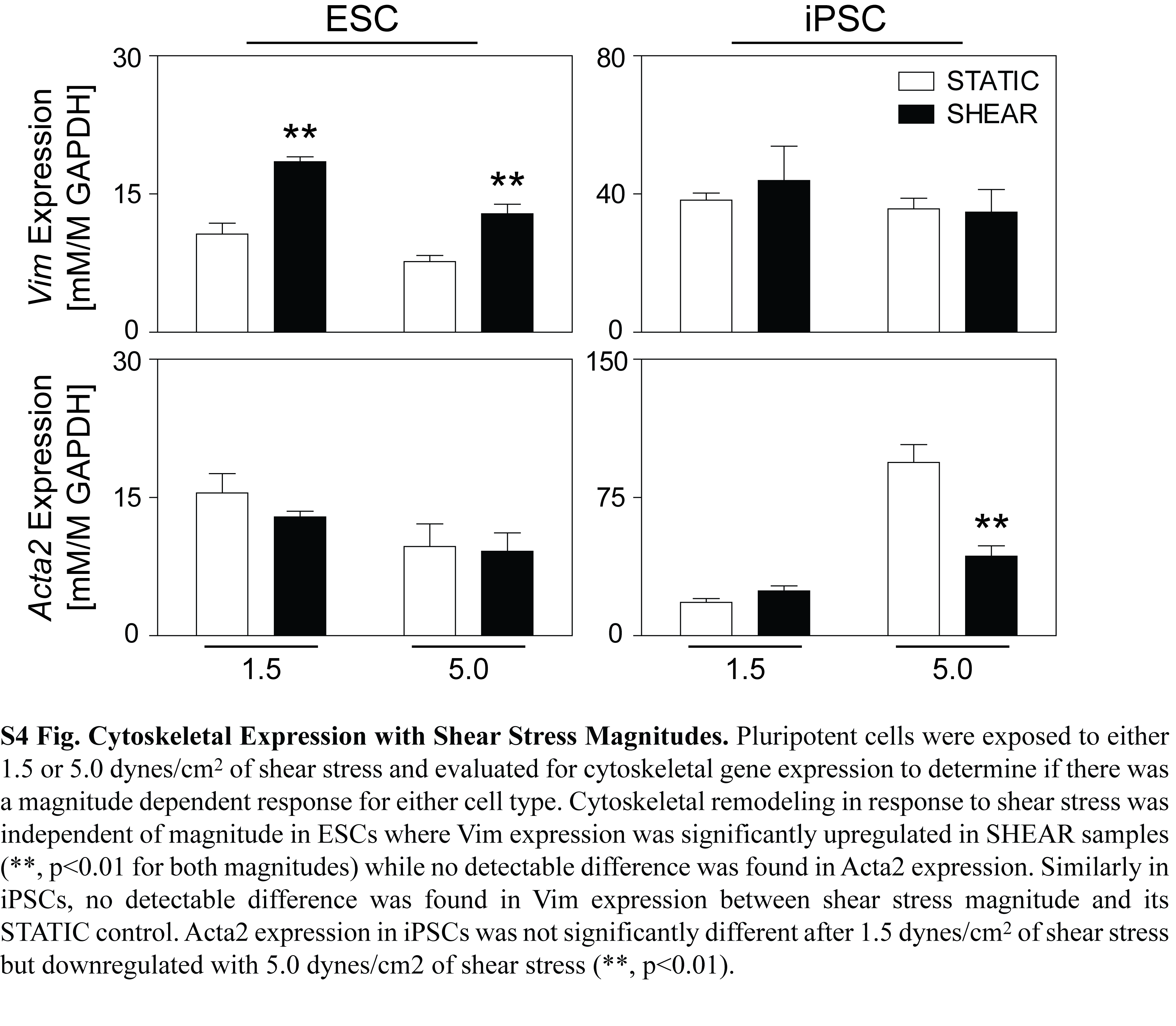

Supplement: S4 Fig — Pluripotent cells were exposed to either 1.5 or 5.0 dynes/cm2 of shear stress and evaluated for cytoskeletal gene expression to determine if there was a magnitude dependent response for either cell type. Cytoskeletal remodeling in response to shear stress was independent of magnitude in ESCs where Vim expression was significantly upregulated in SHEAR samples (p<0.01 for both magnitudes) while no detectable difference was found in Acta2 expression. Similarly in iPSCs, no detectable difference was found in Vim expression between shear stress magnitude and its STATIC control. Acta2 expression in iPSCs was not significantly different after 1.5 dynes/cm2 of shear stress but downregulated with 5.0 dynes/cm2 of shear stress (**, p<0.01). (TIF) [file pone.0145084.s004.tif]
